# Surgical removal of implanted microchips to correct MRI susceptibility artifacts in mice

**DOI:** 10.1002/mrm.70003

**Published:** 2025-07-22

**Authors:** Elizabeth Hipskind, Nicole Hernandez, Sydney Fox, Tina Manirambona, Rita Schack, Brian Gibson, Robia G. Pautler

**Affiliations:** ^1^ Department of Neuroscience Baylor College of Medicine Houston Texas USA; ^2^ Center for Comparative Medicine Baylor College of Medicine Houston Texas USA; ^3^ Department of Integrative Physiology Baylor College of Medicine Houston Texas USA

**Keywords:** mice, microchip, MRI, preclinical model, surgical removal, susceptibility artifact

## Abstract

**Purpose:**

Implanted microchips are becoming increasingly common in research for animal identification and have been adopted by commercial vendors for some mouse strains. However, they often contain metal components, which generate magnetic susceptibility artifacts on MR images. Despite this, some microchips are marketed as MR‐compatible, even though they are likely to affect image quality.

**Methods:**

We assessed the impact of a radiofrequency identification microchip on MR images of the mouse brain and present a method for precise surgical removal. A handheld magnet was used to locate and stabilize the microchips during removal. Mice were imaged before and after microchip removal.

**Results:**

Although marketed as MR‐compatible, implanted microchips caused magnetic susceptibility artifacts in all imaged mice, despite variation in the location of the microchip. Surgical removal corrected these artifacts, allowing for high‐resolution imaging without interference. All subjects recovered well from the procedure.

**Conclusion:**

The discrepancy in the use of the term “MR‐compatible” highlights a disconnect between researchers and some manufacturers. Microchips should be carefully evaluated for experiments involving MRI. In the event that microchips require removal before imaging, surgical removal using a handheld magnet to precisely locate the microchips is effective.

## 1 INTRODUCTION

MRI is a noninvasive approach for disease diagnosis, staging, monitoring, and more. Preclinical MRI has emerged as a powerful tool for translational research, as many of the same methods can be used in both animal models and human patients. A search in PubMed for preclinical studies in mice (search term “MRI” AND “MOUSE,” performed on Feb. 12, 2025) returned 19,975 results, highlighting the impact of preclinical MRI. However, both humans and mice can have implants that may affect MR imaging. Indeed, microchipping has recently been adopted by commercial vendors of mouse models of human disease.

Implanted microchips are an increasingly common method of animal identification for research purposes. Compared with ear tags or ear punches, they are less likely to be lost or obscured over time. The microchips can be programmed to store information such as a subject identifier, genotype, or other group information. Most radiofrequency identification (RFID) microchips do not require a battery, making them ideal for long‐term use.

Although there are a number of benefits to using RFID chips, they can interfere with some types of experiments, including MRI. These microchips include metal components, which can introduce susceptibility artifacts when imaging.^1^, ^2^, ^3^, ^4^ Susceptibility artifacts result when materials with differing magnetic susceptibility distort the local magnetic field in the MRI environment.^5^, ^6^ The pattern of interference depends on the location of the chip relative to the imaging planes, the amount and type of metal used, and the scan type.^2^, ^6^, ^7^

Although the formal labeling of devices as MR‐compatible is no longer in use, the term has been used to refer to items that do not negatively affect MR image quality.^8^, ^9^, ^10^, ^11^

Here, we demonstrate an RFID microchip marketed as MR compatible resulted in susceptibility artifacts on MR images of the mouse brain, including a quantification using B_0_ maps. We then describe a simple surgical method of microchip removal for mice that are difficult to replace. We also discuss the nuance between MR‐safe and MR‐compatible. Finally, we discuss options to avoid microchips for preclinical MRI experiments.

## 2 METHODS

All experiments were conducted with the approval of the Institutional Animal Care and Use Committee at Baylor College of Medicine in accordance with the National Institutes of Health *Guide for the Care and Use of Laboratory Animals*.

Mice (*n* = 24) were purchased from the Jackson Laboratory and came implanted with UID MiniMax Programmable Microchips (UID Identification Solutions, Lake Villa, IL, USA). Microchips were implanted between the shoulder blades at approximately 2 weeks of age by the vendor. The chips are 1.2 × 8.3 mm and are coated with biocompatible glass. At the time of purchase, microchip documentation described them as “MRI compatible.”^12^

### 2.1 MRI

Mice were approximately 6 months of age at the time of imaging. All MR images were acquired on a 9.4T Bruker advance NEO  20‐cm‐bore MRI using Paravision 360 software. Mice were initially anesthetized with 2%–3% isoflurane and secured in the scanning bed, then maintained on 1%–2% isoflurane. Heart rate, respiration, and temperature were monitored throughout imaging using a small‐animal monitoring system (SA Instruments, Inc., Stony Brook, NY, USA). A localizer scan was run first, where the susceptibility artifacts were initially detected, using repetition time (TR) = 100 ms, echo time (TE) = 2.3 ms, slice thickness = 1 mm, number of slices = 1, field of view (FOV) = 40 × 40 mm, matrix = 256 × 256, and spatial resolution = 0.156 mm/pixel. Artifacts were replicated in a total of 8 mice (4 males, 4 females). Two additional mice without microchips were imaged to rule out an equipment issue. Next, a T2_TurboRARE scan was used to further characterize the imaging artifacts, with TE = 33 ms, TR = 2500 ms, slice thickness = 0.7 mm, number of slices = 9, FOV = 30 × 20 mm, image size = 256 × 256, and spatial resolution = 0.117 mm/pixel. These scans were repeated for all mice following microchip removal. An additional B_0_ field map was collected before and after removal of the microchip, using TR = 20 ms, TE = 3.569 ms, flip angle = 30°, slice thickness = 30 mm, number of slices = 1, FOV = 30 × 30 mm, matrix = 64 × 64, and spatial resolution = 0.469 mm/pixel. To quantify the B_0_ map, the results were scaled to 256, and signal intensity was extracted from the image using *ImageJ*.^13^

### 2.2 Implant removal

The microchips were initially located using a handheld magnet (Craftsman model CMMT98316), and the locations were recorded for each mouse to facilitate surgical preparation. To prepare the surgical site, the subject was anesthetized with isoflurane via precision vaporizer. A magnet was used to locate the implant (dorsal scapula to lateral right or left neck or thorax), and the surgical site was aseptically prepared in the following manner: The hair was clipped; a depilatory cream was applied; the area was wiped with three alternating applications of betadine scrub and alcohol; and the patient was draped.

A magnet (sterilized pre‐operatively using ethylene oxide) was used to lift the microchip, where it was grasped with forceps or micro‐mosquito forceps. A small incision, about 1–2 mm, was made with a 15‐blade scalpel over one pole of the microchip. The microchip was then retracted from the incision. The skin was closed with tissue adhesive when > 1‐mm defect was observed. The patient was recovered from anesthesia. The average time each mouse was under anesthesia was approximately 8 min. Following microchip removal, at least 2 weeks elapsed before imaging was resumed.

## 3 RESULTS

### 3.1 Microchips cause susceptibility artifacts on MRI images

**FIGURE 1:**
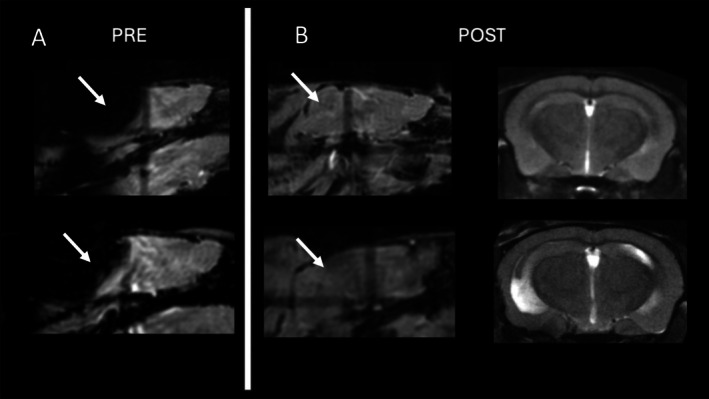
Representative mouse brain images taken before (A) and after (B) microchip removal. Arrows show the same location with and without susceptibility artifacts. Sagittal images from localizer scans; axial images from T2_TurboRARE scans.

Susceptibility artifacts were noted on all eight of the imaged mice. All microchips were positioned with the long side parallel to the body axis and therefore positioned along the bore of the magnet. The severity of the artifacts depended on the location of the microchip, with implants located on the dorsal scapula causing more extensive artifacts. Before microchip removal, susceptibility artifacts caused large‐scale signal loss across the posterior half of the brain (Figure 1A). It was impossible to obtain adequate shimming on the mice with microchips, and we were able to obtain half‐height water peak linewidths of 75 to greater than 100 Hz. For this reason and because of the presence of the significant susceptibility artifacts, imaging was stopped after the localizer scan. To rule out an equipment issue, two additional mice without microchips were imaged, and these scans did not show the susceptibility artifacts. No adverse clinical effects were noted in the microchipped mice that underwent MRI.

Following microchip removal, the susceptibility artifacts were corrected, and we proceeded with higher resolution scanning (Figure 1B).

### 3.2 Characterizing changes in the local magnetic field

**FIGURE 2:**
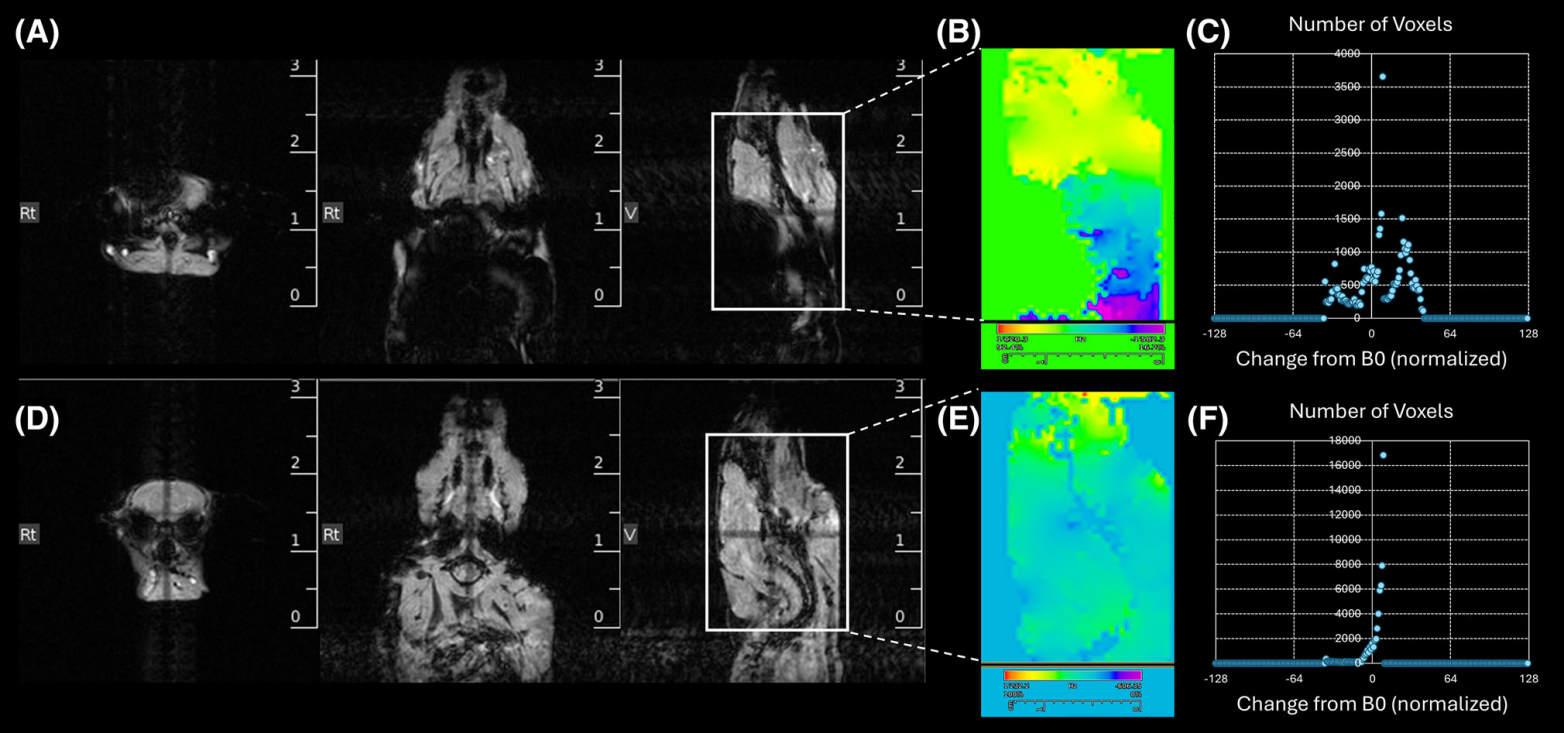
B_0_ field maps before and after microchip removal. (A) Reference scan with axial, coronal, and sagittal views showing the magnetic susceptibility artifact before microchip removal. (B) Associated B_0_ field map depicting magnetic field inhomogeneity. Scale bar represents change in resonant frequency (Hz) from B_0_. (C) Quantification of the normalized field change in the brain area, where B_0_ is set to 0. (D–F) After microchip removal, the artifact is not apparent on reference scan (D), with more uniform B_0_ map (E) and quantification of normalized change from B_0_ (F).

We used a B_0_ field map to assess how the implanted microchips contribute to local inhomogeneities in the magnetic field. Before microchip removal, signal loss is evident on the localizer scan (Figure 2A). On the corresponding B_0_ map, there are widespread local field inhomogeneities, with abrupt changes within the brain (Figure 2B). Next, field changes for each voxel were scaled in *ImageJ* ^13^ and extracted from the brain area (Figure 2C). A more uniform B_0_ map will have a distribution centered around 0 after scaling. In Figure 2C, there are distinct peaks both above and below B_0_, indicating local field changes. Following microchip removal, the artifact is removed (Figure 2D), and the variability in the field map is decreased (Figure 2E). Figure 2F shows the scaled change from B_0_ for each voxel, with most voxels near B_0_.

### 3.3 Surgical removal of microchips

**FIGURE 3:**
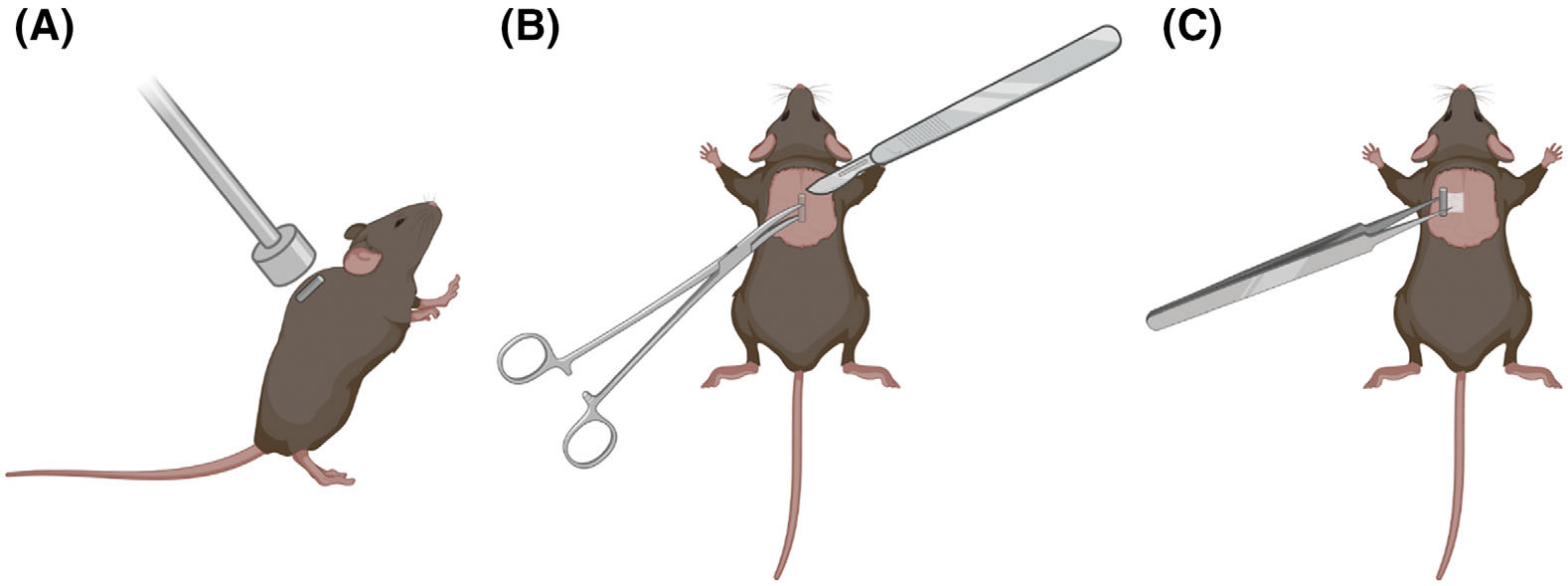
Surgical removal of implanted microchips aided by a handheld magnet. (A) Locating the implanted microchip using a handheld magnet. (B) Following surgical prep, a sterile magnet was used to attract the chip through the skin, so it may be grasped with forceps. A scalpel was used to make a small incision over one pole of the microchip. (C) Variable amounts of connective tissue were observed attached to the microchips. Created in BioRender. Hernandez, N. (2025) https://BioRender.com/p54k620.

First, we used a handheld magnet to precisely locate the microchips (Figure 3A). Although the microchips were initially implanted subcutaneously between the shoulder blades by the vendor, some migration was observed before fixation by connective tissue. There were no obvious swellings or palpable masses detected in the area of the implant before removal. Of the 24 mice, 13 (54%) microchips were found between the scapulae, 6 (25%) were found on the left side (lateral left neck), and 5 (21%) were found on the right side (lateral right neck to right thorax). Initial efforts to locate the microchips without a magnet failed due to the variability in location. All microchips were successfully removed surgically (Figure 3B). The use of a sterilized handheld magnet significantly increased our ability to locate and stabilize the microchips during removal.

We observed varying levels of fibrous connective tissue surrounding the implant (Figure 3C). In one case, the end of the implant was slightly crushed with the forceps while trying to retract it. No glass was observed in the surgical site, but caution is advised to prevent damaging glass‐coated microchips.

## 4 DISCUSSION

We observed susceptibility artifacts on brain images due to implanted microchips in laboratory mice, and these artifacts were corrected after surgical microchip removal.

RFID microchips respond to a radiofrequency (RF) pulse from a handheld or tabletop reader system. The RF pulse is detected by an antenna and generates an electrical current within the microchip, causing it to emit a signal containing the programmed information. However, these microchips use metal components and can contribute to susceptibility artifacts in MRI images that extend well beyond the implant itself.

Susceptibility artifacts increase as the MRI field strength increases.^5^, ^14^, ^15^ Given the small size of the mouse and the rise of ultrahigh‐field imaging, implanted microchips are likely to disrupt a wide range of preclinical applications. Here, we showed susceptibility artifacts on brain images from microchips implanted in the thoracic region. In addition to the neuroscience field, this phenomenon would likely affect imaging studies in a wide range of areas, including cardiopulmonary applications. Even in much larger animals, such as dogs and cats, implanted microchips can render scans undiagnostic in a veterinary setting.^1^ For mice, the microchips are much larger relative to body size; therefore, more of the animal is affected by imaging artifacts.

Magnetic susceptibility artifacts occur any time there is an abrupt shift in the magnetic susceptibility of adjacent materials, which causes local inhomogeneities in the magnetic field.^5^, ^6^ This occurs naturally in the body between biological materials such as interfaces between bone and tissue, and techniques to minimize these artifacts have been studied extensively.^16^, ^17^, ^18^, ^19^, ^20^, ^21^, ^22^ However, the magnetic susceptibility of metallic implants is much higher, rendering mitigation strategies insufficient,^23^ particularly for the small body size of the mouse and high field strengths used in preclinical imaging.

A B_0_ field map provides an estimate of the local deviation from the main magnetic field (B_0_). The implanted microchips caused deviations above and below B_0_ that extended into the brain, which were corrected after microchip removal. The B_0_ field map can be used for shimming as well as artifact correction during postprocessing.^24^ Certain scan types are particularly vulnerable to artifacts due to variation in B_0_, including echo‐planar imaging and MR spectroscopy. The particular scan type and research question should be considered to evaluate whether artifact correction methods will be sufficient or whether surgical removal is warranted. Here, we found that the microchip contributes to abrupt shifts in B_0_, and shimming was insufficient to correct the magnetic susceptibility artifact.

Saito et al. evaluated susceptibility artifacts and tissue injury from implanted microchips in dogs. They found that microchips interfered with imaging of the spinal cord but did not find implant migration or clinically significant tissue damage.^2^ This study was conducted at 1.5 T. In high magnetic fields used for preclinical research, there is increased potential for implant heating or migration. Although documentation for the UID MiniMax chips says they have been tested up to 12 T with no migration or heat injury,^12^ we found that handheld magnets are able to pull on the implant and lift the surrounding skin, suggesting the possibility of tissue injury or pain in an MRI environment with high magnetic fields. This could also potentially affect studies that image awake mice.

A communication gap between the MRI community and some manufacturers may contribute to issues involving susceptibility artifacts. In this case, the microchips were marketed as “compatible with MRI, X‐ray, Ultrasound, and NMR.”^25^ “MR safe” is formally defined by the American Society for Testing and Materials as “an item that poses no known hazards resulting from exposure to any MR environment. MR Safe items are composed of materials that are electrically nonconductive, nonmetallic, and nonmagnetic.”^10^ This is the standard definition adopted by the Food and Drug Administration.^11^ Historically, “MR compatible” referred to items that are MR safe and do not significantly affect image quality, but this definition is no longer in formal use due to the dependence factors such as field strength and scan parameters.^10^ Despite no longer being used, the term “MR compatible” has persisted in the scientific literature, even in articles pertaining to implanted devices in humans.^26^ The microchips described in this report contain ferrous material and react to a small handheld magnet, raising concerns about their suitability for MRI use even in areas that would not be impacted by susceptibility artifacts.^27^ Although the UID MiniMax microchip is intended for use in laboratory animals and therefore is not subject to the labeling standards of the Food and Drug Administration, the description as “compatible for use with MRI” highlights an ongoing disconnect between researchers and manufacturers regarding MRI terminology and artifact concerns.

Given the issues described here regarding susceptibility artifacts in a research‐use microchip marketed as MRI compatible, we recommend verifying that microchips used in preclinical studies do not interfere with the desired imaging before commencing experiments. When mice are already implanted with microchips, surgical microchip removal may be a feasible option if artifacts interfere with MRI. To avoid this scenario altogether, one can contact vendors in advance to request that mice intended for MRI studies do not receive a microchip implant. This request must be submitted well ahead of time; for example, the Jackson Laboratory implants microchips when the mice are approximately 2 weeks old or at the time of genotyping.

In summary, we have demonstrated here that implanted RFID microchips can cause significant imaging artifacts, and surgical removal using a handheld magnet effectively resolves the microchip‐associated susceptibility artifacts.
